# Comparative Analysis of Genioplasty Versus Hyaluronic Acid Injection for Chin Retrusion in Asian Chinese Female Patients: A One‐Year Outcome Evaluation Combining Eye‐Tracking and Scale Assessment

**DOI:** 10.1111/jocd.71117

**Published:** 2026-08-03

**Authors:** Xinyi Gao, Xianglong Zu, Chi Zhang, Jingping Shi, Zexiong Cheng

**Affiliations:** ^1^ Department of Maxillofacial Surgery Nanjing Stomatological Hospital, Medical School of Nanjing University Nanjing People's Republic of China; ^2^ Department of Burn and Plastic Surgery The First Affiliated Hospital of Nanjing Medical University Nanjing People's Republic of China; ^3^ Department of Orthodontics The Affiliated Stomatological Hospital of Nanjing Medical University Nanjing People's Republic of China

**Keywords:** chin retrusion, eye‐tracking analysis, facial aesthetic evaluation, genioplasty, hyaluronic acid injection

## Abstract

**Objective:**

This study compared 1‐year postoperative visual attention patterns and lay‐rated aesthetic outcomes after genioplasty and hyaluronic acid (HA) injection for moderate‐to‐severe chin retrusion in Asian Chinese women.

**Methods:**

Sixteen female patients with comparable baseline severity according to the China (Allergan) Chin Retrusion Scale (CACRS) were included, with eight patients in the genioplasty group and 8 in the HA injection group. Forty‐six lay observers assessed standardized 12‐month postoperative facial photographs in frontal, lateral, and 45° oblique views. Eye‐tracking was used to quantify fixation duration across predefined facial regions, and the Merz Aesthetic Rating Scales (MARS) were used for structured postoperative aesthetic assessment.

**Results:**

The HA injection group showed longer fixation durations in the chin region across all three views, whereas the genioplasty group showed relatively longer fixation durations in the perioral region in the frontal view and in the periorbital region in the 45° oblique view. MARS assessment showed lower postoperative scores for symmetry and contour in the genioplasty group, with no significant between‐group difference in skin quality.

**Conclusions:**

At 1 year postoperatively, genioplasty was associated with lower MARS scores for symmetry and contour and a less chin‐focused fixation pattern compared with HA injection. However, the observed fixation differences were modest and should be interpreted cautiously. Eye‐tracking analysis combined with MARS may provide a quantifiable framework for evaluating postoperative facial aesthetic perception.

## 1 Introduction

Chin morphology plays an important role in facial harmony and overall aesthetic appearance [1]. An ideal chin enhances facial three‐dimensionality and balance, whereas chin retrusion, a common aesthetic concern, often leads to facial disproportionality and may negatively affect facial attractiveness. Beyond aesthetics, chin retrusion can cause psychological distress. Patients often feel self‐conscious due to the attention it draws, leading to discomfort [2]. Among Asian populations, the incidence of mandibular retrusion is relatively high, which is closely associated with the anatomical characteristics of Asian facial skeletal structures [3, 4], typically characterized by flatter bone contours, making chin retrusion more prevalent in this demographic. As society develops and aesthetic expectations rise, the treatment options for addressing chin retrusion have become more diverse. Currently, genioplasty and hyaluronic acid (HA) injections are the primary clinical approaches [5].

Genioplasty involves the surgical reshaping of the chin bone, providing a definitive and permanent correction with stable long‐term outcomes [6]. In contrast, HA injection is a non‐surgical cosmetic procedure that enhances chin volume using hyaluronic acid fillers. This method has gained popularity in recent years due to its simplicity and minimal recovery time. Despite the variety of available treatments, comparative studies evaluating different therapeutic options remain scarce. This research gap has contributed to the lack of standardized clinical guidance, leaving physicians without clear evidence‐based criteria for treatment selection and potentially affecting patient outcomes and satisfaction [7]. Therefore, in‐depth comparative studies on the efficacy and aesthetic outcomes of different treatments are needed to improve patient care and support clinical decision‐making.

Previous studies have evaluated early complications and patient satisfaction associated with genioplasty and chin implants [8], but these have predominantly focused on treatment safety and subjective patient feedback. Despite the importance of chin retrusion correction, a notable gap persists in targeted research on its aesthetic outcomes, especially concerning the long‐term effects of different treatments on chin aesthetics. Because aesthetic outcome assessment is a critical indicator of treatment success, addressing this gap is essential for informing clinical practice. Fortunately, a validated assessment scale for Asian chin projection is available to facilitate these evaluations.

To further bridge this gap, this study used eye‐tracking technology to quantitatively assess postoperative visual attention patterns associated with different correction methods [9, 10, 11, 12]. Eye‐tracking is a research tool that tracks eye position and movement in real time. By quantifying observers' gaze patterns while viewing facial images, it provides quantitative data on the distribution of visual attention [13, 14, 15, 16]. This approach allows postoperative facial aesthetic perception to be examined from a visual attention perspective.

This study hypothesized that, at 1 year after treatment, Asian Chinese women with moderate‐to‐severe chin retrusion who underwent genioplasty would demonstrate different visual attention patterns and MARS assessment outcomes compared with those treated with HA injection. Outcomes were assessed by measuring visual attention distribution using eye‐tracking technology and by evaluating chin symmetry and contour using the MARS scale.

The aim of this study was to quantitatively compare postoperative visual attention patterns and lay‐rated aesthetic outcomes between these two treatment methods and provide preliminary evidence for treatment evaluation in this population.

## 2 Materials and Methods

### 2.1 Study Materials and Participant Images

This study examined postoperative facial images of 16 female patients, aged 19–40 years (mean age 26.0 ± 4.84 years), who presented with chin retrusion. Baseline chin retrusion severity was assessed using the China (Allergan) Chin Retrusion Scale (CACRS), a 5‐point photonumeric scale developed from the original Allergan Chin Retrusion Scale and validated for Chinese subjects [17, 18]. The CACRS grades chin retrusion from 0 to 4, with higher scores indicating greater retrusion. Patients with CACRS scores ≥ 3 were considered to have moderate‐to‐severe chin retrusion and were eligible for inclusion.

Baseline chin retrusion severity was obtained from retrospective clinical documentation and is reported in aggregate form to describe baseline comparability between the two groups. The present eye‐tracking experiment was based on standardized 12‐month postoperative photographs; therefore, pre‐treatment images were not included in the visual assessment. Baseline CACRS data are reported in aggregate form in Table 1.

**TABLE 1:** Baseline characteristics of patients.

Variable	Genioplasty group (*n* = 8)	HA injection group (*n* = 8)	*p*
Age, years	26.5 ± 5.1	25.6 ± 4.8	0.720
Baseline CACRS score	3.5 (3.0–4.0)	3.5 (3.0–4.0)	0.610
CACRS Grade 3, *n* (%)	4 (50.0%)	3 (37.5%)	1.000
CACRS Grade 4, *n* (%)	4 (50.0%)	5 (62.5%)	1.000

*Note:* Data are expressed as mean ± SD, median (interquartile range), or *n* (%), as appropriate.

Abbreviations: CACRS, China (Allergan) Chin Retrusion Scale; HA, hyaluronic acid.

Patients were randomly selected from the retrospective clinical records of the Affiliated Stomatological Hospital of Nanjing Medical University. Between January 2023 and December 2024, 87 female patients with moderate‐to‐severe chin retrusion, defined as a CACRS score ≥ 3, underwent either genioplasty or HA injection and were identified from these records.

For the present eye‐tracking image‐based analysis, available cases were retrospectively reviewed to reduce potential visual attention confounding unrelated to chin morphology. Patients were excluded if they had incomplete 12‐month follow‐up photographs, clearly non‐standardized photographic views, obvious facial scars, marked facial asymmetry, visible inflammatory skin lesions, prominent periorbital or perioral abnormalities, or previous facial aesthetic procedures other than the index chin treatment. Dentoskeletal characteristics were assessed mainly through routine preoperative clinical examination, facial profile evaluation, and occlusal screening; patients with obvious dentofacial deformity, severe skeletal Class II malocclusion, active orthodontic treatment, or those requiring orthognathic surgery were excluded. Among eligible cases with standardized frontal, true lateral, and 45° oblique 12‐month postoperative photographs, 16 patients were randomly selected using a random number table, including eight patients in the genioplasty group and eight patients in the HA injection group.

In this study, horizontal sliding genioplasty with bony advancement, which is widely regarded as a standard approach for correcting chin retrusion, was performed in the surgical group. To standardize the procedure, the degree of advancement was determined according to each patient's baseline CACRS score and clinical assessment.

To evaluate long‐term aesthetic outcomes, standardized frontal, true lateral, and 45° oblique view images were selected from clinical archives. All images were obtained 12 months postoperatively and included postoperative photographs from both the genioplasty and hyaluronic acid (HA) injection groups [4, 19].

### 2.2 Treatment Procedures

All procedures were performed by the same senior attending surgeon with more than 10 years of experience in facial aesthetic surgery to ensure procedural consistency and minimize operator‐related bias.

#### 2.2.1 Genioplasty Group

Patients in the surgical group underwent horizontal sliding genioplasty through an intraoral vestibular approach under general anesthesia. A horizontal osteotomy was performed in the mandibular symphysis region, and the distal bony segment was advanced anteriorly according to the severity of chin retrusion and preoperative aesthetic planning. The mean advancement distance was approximately 6–8 mm. The osteotomized segment was stabilized using titanium mini‐plates and screws. Standard postoperative management included prophylactic antibiotics, chlorhexidine mouth rinses, and a soft diet for 2 weeks. All patients completed routine follow‐up examinations during the postoperative period.

#### 2.2.2 Hyaluronic Acid Injection Group

Patients in the HA injection group underwent chin augmentation using a high G′ cross‐linked hyaluronic acid filler (Juvederm Volux, Allergan Aesthetics, Irvine, CA, USA). The filler was injected into the supraperiosteal plane of the pogonion and adjacent prejowl regions using a 27‐gauge needle. A combination of bolus injection and linear threading techniques was employed to optimize chin projection and contour. The mean injection volume was 1.8 ± 0.4 mL per patient. All injections were performed according to a standardized protocol.

### 2.3 Observer Recruitment

Observers were recruited through community posters and online voluntary registration. Stratified sampling was used to ensure gender balance and representativeness of the general population. A total of 46 community volunteers (23 males and 23 females), aged 19–55 years (mean age 32 years), were recruited as observers. All observers were Asian Chinese. To ensure that the evaluations reflected lay perceptions and were free from professional bias, all participants were required to have no medical background, no employment history in cosmetic medicine, no personal history of chin surgery, and normal visual acuity or corrected visual acuity of at least 1.0. Participants with severe visual impairments were also excluded.

### 2.4 Study Design and Procedure

This comparative study used retrospectively selected, standardized postoperative photographs and prospectively collected eye‐tracking and MARS assessments. The experimental design and observer data collection were pre‐planned to allow for a quantifiable comparison of postoperative visual attention patterns and lay‐rated aesthetic outcomes following genioplasty and hyaluronic acid (HA) injection.

The experimental protocol for the recruited observers encompassed three consecutive phases: eye‐tracking analysis, standardized rater training, and MARS Scale Assessment.

During the eye movement recording phase, participants viewed standardized facial images presented in random order. An integrated eye‐tracking system recorded their visual scanning patterns in response to frontal, lateral, and 45° oblique facial views.

After data acquisition, all participants received standardized training on the Merz Aesthetic Rating Scales (MARS). This calibration session was implemented before the formal evaluation to minimize inter‐rater variability and ensure consistent interpretation of the assessment criteria.

In the final MARS assessment phase, the trained participants systematically evaluated chin aesthetics, assigning scores across the five MARS domains according to established guidelines.

### 2.5 Eye Movement Analysis

**FIGURE 1:**
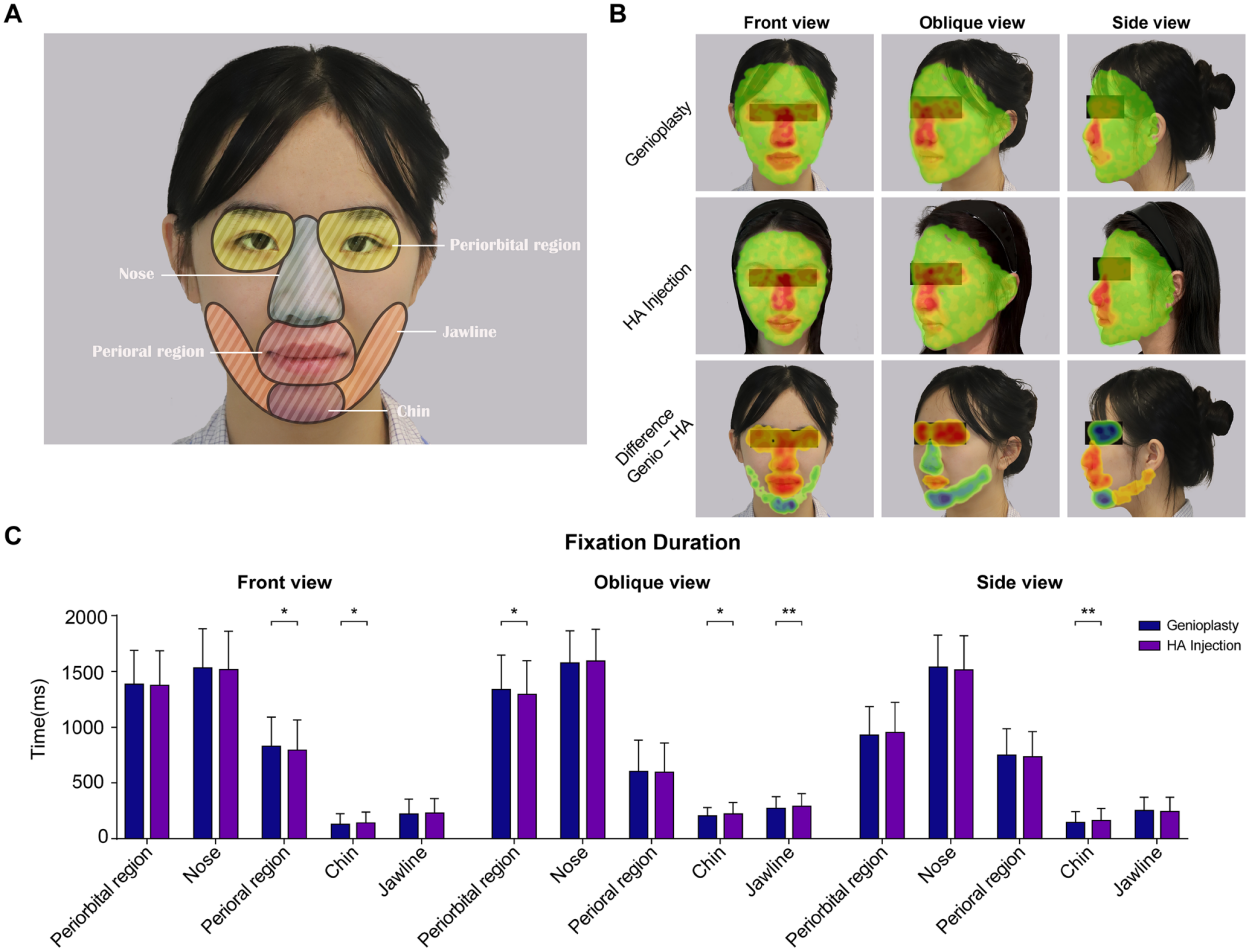
(A) Five predefined facial areas of interest examined in this study. (B) Mean eye movement heat maps for images from the genioplasty and HA injection groups across frontal, 45° oblique, and lateral views, with illustrative difference heat maps shown in the third row. Difference values were defined as genioplasty minus HA injection. For the original heat maps, warmer colors indicate regions receiving more fixations, whereas cooler colors indicate regions receiving fewer fixations. For the illustrative difference heat maps, warmer colors indicate relatively positive values, whereas cooler colors indicate relatively negative values. These difference heat maps are intended as schematic visualizations and should be interpreted together with the AOI‐based quantitative fixation duration analysis. (C) Fixation durations for key facial regions in the Genioplasty and Hyaluronic Acid Injection groups across three viewing angles. Asterisks indicate statistically significant between‐group differences: *p < 0.05; **p < 0.01.

Eye movement data were collected using a Tobii Pro Nano binocular eye tracker (Tobii Pro AB, Stockholm, Sweden) integrated with a 27‐in. display. The system's 60 Hz sampling frequency ensured precise measurements. To eliminate visual aftereffects and ensure the independence of each observation, a 2‐s blackout interval separated the presentation of each facial image. For quantitative analysis, each facial image was divided into five predefined areas of interest (AOIs): the periorbital, nasal, perioral, jawline, and chin regions (Figure 1A). The primary metric analyzed was the cumulative fixation duration within each AOI during the 7‐s stimulus exposure. Blink and saccade artifacts were systematically identified and excluded from subsequent analysis.

### 2.6 MARS Scale Assessment

A comprehensive aesthetic assessment was performed using the validated Merz Aesthetic Rating Scales (MARS). This scale evaluates five domains on a five‐point scale (0–4): Position (chin‐facial harmony), Symmetry (bilateral congruence), Proportion (dimensional harmony), Contour (profile definition), and Skin Quality (textural and pigmentary characteristics). The scoring criteria are as follows: 0 represents ideal aesthetics, 1 indicates minimal defects not requiring treatment, 2 signifies mild defects possibly needing correction, 3 denotes noticeable defects requiring treatment, and 4 represents severe defects necessitating intervention. To ensure consistent ratings, all evaluators underwent standardized MARS training between eye movement recording and formal assessment. All evaluations were conducted in strict accordance with the MARS framework.

### 2.7 Ethical Approval and Study Timeline

This study was conducted from January to September 2025 and was approved by the Ethics Committee of Nanjing Medical University (approval number: PJ2025‐125‐001). All procedures were performed in accordance with the principles of the Declaration of Helsinki. Written informed consent was obtained from all patients whose images were used and from all observers who participated in the eye‐tracking and MARS assessments.

To protect participant privacy and ensure data security, all facial images were anonymized by removing personal identifiers and retaining only serial numbers. Furthermore, all eye‐tracking data and associated personal information were stored in an encrypted format, and access was strictly limited to authorized core research team members to prevent data breaches or misuse.

### 2.8 Data Analysis

Statistical analyses were performed using SPSS Statistics version 27.0 (IBM Corp., Armonk, NY, USA). Continuous variables were tested for normality. Normally distributed continuous variables are presented as mean ± standard deviation and were compared using independent‐samples *t*‐tests. Ordinal variables are presented as median and interquartile range and were compared using the Mann–Whitney *U* test. Categorical variables are presented as number and percentage and were compared using Fisher's exact test.

For baseline comparisons, age was compared between groups using an independent‐samples *t*‐test. Baseline CACRS scores were compared using the Mann–Whitney *U* test, and CACRS grade distributions were compared using Fisher's exact test.

Inter‐rater reliability of MARS assessment was evaluated using intraclass correlation coefficients, including single‐measure and average‐measure ICCs. For postoperative eye‐tracking and MARS outcomes, between‐group comparisons were performed using independent‐samples *t*‐tests, and between‐group differences were reported with 95% confidence intervals where appropriate. A two‐sided *p* value of < 0.05 was considered statistically significant.

## 3 Results

### 3.1 Baseline Characteristics

**FIGURE 2:**
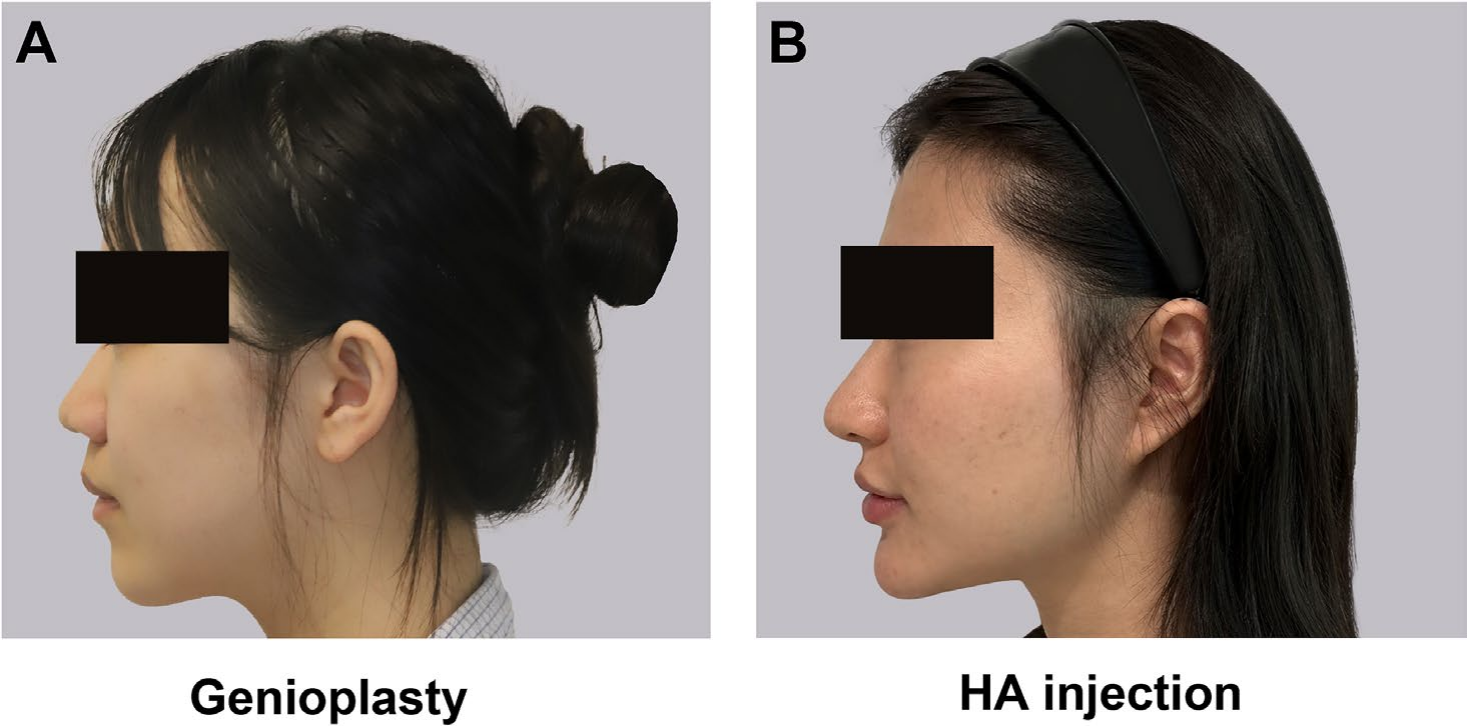
Representative standardized 12‐month postoperative true lateral images of the two treatment groups. (A) genioplasty group. (B) HA injection group. These images are provided as visual examples only and do not represent paired comparisons.

The baseline characteristics of the two groups are shown in Table 1. There was no significant difference in age between the genioplasty and HA injection groups (26.5 ± 5.1 years vs. 25.6 ± 4.8 years, *p* = 0.720). Baseline chin retrusion severity was also comparable between groups, with a median CACRS score of 3.5 (3.0–4.0) in both groups (*p* = 0.610). The distribution of CACRS Grade 3 and Grade 4 patients did not differ significantly between groups (both *p* = 1.000). These findings indicate that the two groups were broadly comparable in baseline chin retrusion severity before treatment. Representative postoperative images from the two treatment groups are shown in Figure 2 as visual examples.

### 3.2 Fixation Duration

During the 7‐s visual stimulus period, several facial regions showed statistically significant between‐group differences in fixation duration. The 95% confidence intervals were calculated as the between‐group difference between the genioplasty group and the HA injection group.

The HA injection group showed longer fixation durations on the chin region than the genioplasty group across all three views. In the frontal view, chin fixation duration was 142 ± 80 ms in the genioplasty group and 156 ± 82 ms in the HA injection group, with a 95% CI of −26 to −2 ms (*p* = 0.015). In the 45° oblique view, chin fixation duration was 218 ± 59 ms in the genioplasty group and 235 ± 89 ms in the HA injection group, with a 95% CI of −28 to −6 ms (*p* = 0.030). In the lateral view, chin fixation duration was 161 ± 81 ms in the genioplasty group and 177 ± 92 ms in the HA injection group, with a 95% CI of −29 to −4 ms (*p* = 0.008). Because the confidence intervals were calculated as genioplasty minus HA injection, the negative values indicate longer chin fixation durations in the HA injection group.

In the 45° oblique view, the HA injection group also showed longer fixation duration on the jawline region than the genioplasty group (286 ± 90 ms vs. 304 ± 98 ms, 95% CI −33 to −6 ms, *p* = 0.005). By contrast, the genioplasty group showed longer fixation duration on the perioral region in the frontal view (844 ± 247 ms vs. 807 ± 258 ms, 95% CI 1 to 74 ms, *p* = 0.045) and on the periorbital region in the 45° oblique view (1355 ± 293 ms vs. 1312 ± 285 ms, 95% CI 1 to 85 ms, *p* = 0.042). No significant between‐group differences were observed in the remaining facial regions. These findings indicate modest but quantifiable differences in postoperative visual attention patterns between the two procedures (Table 2, Figure 1B,C).

**TABLE 2:** Comparison of fixation durations in specific facial regions after the two procedures (mean ± SD, ms).

	Facial region	Front view				Oblique view				Side view			
		Genioplasty	HA injection	95% CI	*p*	Genioplasty	HA injection	95% CI	*p*	Genioplasty	HA injection	95% CI	*p*
Fixation duration	Periorbital region	1401 ± 288	1391 ± 296	−32~53	0.623	1355 ± 293	1312 ± 285	1~85	0.042^#^	944 ± 242	967 ± 255	−60 ~ 13	0.2
	Nose	1547 ± 334	1533 ± 328	−34 ~ 62	0.565	1591 ± 273	1607 ± 270	−55~23	0.419	1555 ± 272	1530 ± 291	−16~65	0.234
	Perioral region	844 ± 247	807 ± 258	1~74	0.045^#^	617 ± 270	610 ± 251	−31~44	0.732	763 ± 224	747 ± 213	−16~47	0.331
	Chin	142 ± 80	156 ± 82	−26~−2	0.015^#^	218 ± 59	235 ± 89	−28~−6	0.03^#^	161 ± 81	177 ± 92	−29~−4	0.008^#^
	Jawline	235 ± 119	243 ± 116	−25~9	0.338	286 ± 90	304 ± 98	−33~−6	0.005^#^	266 ± 105	258 ± 113	−8~24	0.305

*Note:* The 95% CI refers to the between‐group difference calculated as genioplasty minus HA injection. Negative values indicate longer fixation duration in the HA injection group, whereas positive values indicate longer fixation duration in the genioplasty group.

Abbreviations: CI, confidence interval; HA, hyaluronic acid.

^#^ *p* < 0.05.

### 3.3 Comparison of MARS Scale Scores

To ensure rating reliability, we assessed the inter‐rater agreement of the MARS scale using intraclass correlation coefficient (ICC) analysis. The single‐measure ICC was 0.51, indicating moderate consistency among individual raters. However, the average‐measure ICC was 0.98 (*p* < 0.001), demonstrating excellent overall agreement among multiple raters. These results confirm the reliability of the MARS scoring method for subsequent analyses.

Table 3 presents the comparison of postoperative MARS scores between the genioplasty and HA injection groups. Lower MARS scores indicate more favorable aesthetic ratings in the corresponding domain. The 95% confidence intervals were calculated as the between‐group difference between the genioplasty group and the HA injection group.

**TABLE 3:** Comparison of postoperative MARS scores between the two procedures (mean ± SD, points).

Assessment dimension	Genioplasty	HA injection	95% CI	*p*
Position	0.80 ± 0.57	0.88 ± 0.68	−0.18~0.01	0.07
Symmetry	0.69 ± 0.51	0.79 ± 0.63	−0.18~−0.01	0.02^#^
Proportion	0.62 ± 0.52	0.69 ± 0.60	−0.16~0.01	0.07
Contour	0.82 ± 0.57	0.92 ± 0.66	−0.19~−0.01	0.03^#^
Skin Quality	0.74 ± 0.50	0.78 ± 0.61	−0.12~0.04	0.32

*Note:* The 95% CI refers to the between‐group difference calculated as genioplasty minus HA injection. Lower scores indicate more favorable aesthetic ratings in the corresponding domain.

Abbreviations: CI, confidence interval; HA, hyaluronic acid; MARS, Merz Aesthetic Rating Scales.

^#^ *p* < 0.05.

The genioplasty group showed lower postoperative MARS scores than the HA injection group for Symmetry (0.69 ± 0.51 vs. 0.79 ± 0.63, 95% CI −0.18 to −0.01, *p* = 0.020) and Contour (0.82 ± 0.57 vs. 0.92 ± 0.66, 95% CI −0.19 to −0.01, *p* = 0.030). Because the confidence intervals were calculated as genioplasty minus HA injection, the negative values indicate lower MARS scores in the genioplasty group for these two domains.

No statistically significant between‐group differences were observed for Position (0.80 ± 0.57 vs. 0.88 ± 0.68, 95% CI −0.18 to 0.01, *p* = 0.070), Proportion (0.62 ± 0.52 vs. 0.69 ± 0.60, 95% CI −0.16 to 0.01, *p* = 0.070), or Skin Quality (0.74 ± 0.50 vs. 0.78 ± 0.61, 95% CI −0.12 to 0.04, *p* = 0.320). These findings suggest that, at 1 year postoperatively, the between‐group differences in lay‐perceived aesthetic ratings were mainly observed in the symmetry and contour domains (Table 3).

## 4 Discussion

This study used eye‐tracking technology and the MARS scale to quantitatively compare postoperative visual attention patterns and lay‐perceived aesthetic ratings after genioplasty and hyaluronic acid injection for chin retrusion at the 1‐year follow‐up. The goal was to provide preliminary evidence regarding postoperative aesthetic perception rather than to establish definitive clinical superiority of either procedure. The results revealed significant differences in both observers' gaze patterns and MARS assessment outcomes between the two procedures. Furthermore, MARS scale scores demonstrated that the procedures had varying effects on different aesthetic dimensions, offering a multidimensional framework for clinical decision‐making [10, 11]. To further explore the underlying reasons for these differences, we incorporated findings from reference [20], which focused on volumetric analysis of lower facial third aesthetic treatments and their relationship with subjective perceptions of attractiveness. The distinct volumetric characteristics of genioplasty and HA injection may partly explain the observed postoperative differences. The postoperative contour after HA injection may be influenced by filler distribution and soft‐tissue behavior over time. In contrast, genioplasty allows for structural adjustment through bony reshaping, which may partly explain the lower postoperative MARS scores for symmetry and contour observed in the genioplasty group. Previous studies have demonstrated associations between visual attention patterns and facial attractiveness assessments. However, eye‐tracking metrics alone cannot determine aesthetic preference and should be interpreted together with complementary assessment tools.

Eye‐tracking technology offers a key benefit by providing quantifiable information regarding visual attention allocation when observers view postoperative facial images [21]. Compared to the genioplasty group, the hyaluronic acid injection group showed significantly longer fixation durations in the chin region from the frontal (156 ± 82 ms vs. 142 ± 80 ms, *p* = 0.015), 45° oblique (235 ± 89 ms vs. 218 ± 59 ms, *p* = 0.030), and lateral views (177 ± 92 ms vs. 161 ± 81 ms, *p* = 0.008), as well as in the jawline region under the 45° oblique view (304 ± 98 ms vs. 286 ± 90 ms, *p* = 0.005). These longer fixation durations indicate that the chin region attracted greater visual attention from observers in the HA injection group. Eye‐tracking metrics provide quantifiable information regarding visual attention allocation; however, they do not directly indicate whether the observed feature is perceived positively or negatively. Therefore, the eye‐tracking findings should be interpreted together with the MARS assessment results. In the present study, the HA injection group demonstrated longer fixation durations in the chin region, whereas the genioplasty group showed lower MARS scores in symmetry and contour. These findings suggest that the two treatment modalities may produce different patterns of visual attention and aesthetic assessment at the 1‐year follow‐up. In contrast, the genioplasty group exhibited significantly longer fixation times on the mouth area in frontal views (844 ± 247 ms vs. 807 ± 258 ms, *p* = 0.045) and on the eye area in 45° oblique views (1355 ± 293 ms vs. 1312 ± 285 ms, *p* = 0.042). These results suggest a different pattern of postoperative visual attention distribution between the two procedures rather than direct evidence of superior aesthetic perception in either group [13, 22, 23]. The different visual attention pattern observed in the genioplasty group may reflect differences in postoperative facial appearance. However, the clinical significance of these gaze patterns requires further investigation using additional aesthetic assessment tools and patient‐reported outcome measures.

The MARS findings complement the eye‐tracking results by providing lay‐perceived ratings across specific postoperative aesthetic domains. In the present study, the genioplasty group showed lower postoperative MARS scores than the HA injection group for Symmetry (0.69 ± 0.51 vs. 0.79 ± 0.63, 95% CI −0.18 to −0.01, *p* = 0.020) and Contour (0.82 ± 0.57 vs. 0.92 ± 0.66, 95% CI −0.19 to −0.01, *p* = 0.030). Because lower MARS scores indicate more favorable aesthetic ratings, these findings suggest that the between‐group differences in lay‐perceived postoperative aesthetic ratings were mainly observed in the symmetry and contour domains.

One possible explanation is the anatomical difference between the two treatment modalities. Genioplasty directly repositions the bony chin segment and may therefore provide a more defined skeletal framework for postoperative chin contour at the 1‐year follow‐up. In contrast, HA injection relies on soft‐tissue augmentation, and its postoperative contour may be influenced by filler distribution and gradual resorption over time. However, the absolute differences in MARS scores were small, and the lower bounds of the confidence intervals were close to zero. Therefore, these findings should be interpreted cautiously and should not be considered definitive evidence of clinical superiority.

No statistically significant between‐group differences were observed for Position (0.80 ± 0.57 vs. 0.88 ± 0.68, 95% CI −0.18 to 0.01, *p* = 0.070), Proportion (0.62 ± 0.52 vs. 0.69 ± 0.60, 95% CI −0.16 to 0.01, *p* = 0.070), or Skin Quality (0.74 ± 0.50 vs. 0.78 ± 0.61, 95% CI −0.12 to 0.04, *p* = 0.320). The nonsignificant results for Position and Proportion indicate that the data do not support a statistically significant between‐group difference in these domains. The lack of a significant difference in Skin Quality may be related to the 1‐year postoperative assessment time point, at which visible procedure‐related skin changes were limited in both groups.

An important consideration when interpreting the present findings is the durability of hyaluronic acid augmentation in the chin region. Traditionally, HA fillers have been regarded as temporary materials that gradually undergo resorption over time. However, Mashiko et al. reported that supraperiosteal or onlay HA injection onto a bony surface may induce a longer‐lasting or semipermanent volumetric effect, possibly related to tissue remodeling around the injected material [24]. Therefore, the 12‐month comparison between genioplasty and HA injection should not be interpreted simply as a comparison between a permanent surgical procedure and a short‐lasting filler treatment.

In the present study, no additional HA injections or touch‐up procedures were performed during the follow‐up period. Thus, the postoperative outcomes of the HA group reflected the persistence of the initial filler treatment at 1 year. Future studies with serial three‐dimensional imaging and longer follow‐up are needed to determine whether the observed chin contour after HA injection represents residual filler volume, tissue remodeling, or both.

Based on these findings, treatment selection should be guided by clinical needs and patient preferences. For patients with moderate‐to‐severe chin retrusion seeking more durable correction, genioplasty may be the more appropriate option because it directly addresses the underlying skeletal deficiency. Hyaluronic acid injection may still be considered in selected patients seeking a less invasive approach, but its variable durability and the potential need for repeat treatment should be clearly explained.

### 4.1 Study Limitations

This study has several limitations. First, the sample size was small (*n* = 16) and limited to Asian Chinese women, which restricts the generalizability of the findings to other ethnicities and genders. In addition, dentoskeletal characteristics were mainly screened through routine preoperative clinical assessment, and comprehensive cephalometric measurements were not available for all patients; therefore, subtle dentoskeletal discrepancies could not be fully quantified. Second, the study only compared genioplasty and HA injection, omitting other common procedures like chin implants and autologous fat grafting. Third, the one‐year follow‐up period was insufficient to assess long‐term stability, particularly for HA injection, given the potential for filler resorption, persistence, or tissue remodeling over time. Fourth, although baseline CACRS data were available to assess baseline comparability, the eye‐tracking and MARS assessments were based only on 12‐month postoperative photographs. Therefore, pre‐to‐postoperative changes in visual attention patterns or aesthetic ratings could not be evaluated. Finally, the study focused mainly on eye‐tracking metrics and lay‐rated aesthetic assessment, without incorporating patient‐reported satisfaction or psychological measures. In addition, the aesthetic ratings were obtained from lay observers rather than expert clinicians. Although this design reflected lay‐perceived aesthetic perception and inter‐rater reliability was assessed using ICC, future studies should include both lay and expert evaluators to compare perceptual differences between these groups. Future research should address these limitations by including larger, more diverse cohorts, comparing a wider range of procedures, extending the follow‐up duration, and incorporating multidimensional assessments that combine objective measures with patient‐reported outcomes.

## 5 Conclusion

This exploratory study provides a 1‐year postoperative comparison of visual attention patterns and lay‐perceived aesthetic ratings after genioplasty and HA injection for moderate‐to‐severe chin retrusion in Asian Chinese women. The two groups were broadly comparable in baseline CACRS severity. At 1 year postoperatively, genioplasty was associated with lower MARS scores for chin symmetry and contour and a less chin‐focused fixation pattern compared with HA injection. However, the absolute differences in fixation duration were modest, and these findings should be interpreted cautiously. The combined use of eye‐tracking analysis and MARS assessment may provide a quantifiable framework for evaluating postoperative facial aesthetic perception. Further studies with larger samples, baseline‐to‐postoperative comparisons, and longer follow‐up are needed to validate these findings.

## Author Contributions

X.G. and X.Z. contributed equally to this work. X.G. designed the study, conducted the eye‐tracking experiments, performed data curation, and wrote the original draft of the manuscript. X.Z. was responsible for patient recruitment, image collection, MARS scale training and assessment, and statistical analysis. J.S. and Z.C. jointly supervised the project and critically revised the manuscript. All authors reviewed and approved the final manuscript.

## Funding

The authors have nothing to report.

## Ethics Statement

The study protocol was reviewed and approved by the Ethics Committee of Nanjing Medical University (approval number: PJ2025‐125‐001). The study was conducted in accordance with the Declaration of Helsinki.

## Consent

Written informed consent was obtained from all patients whose images were used and from all observers who participated in the eye‐tracking and MARS assessments. All participants were fully informed about the study procedures prior to their participation.

## Conflicts of Interest

The authors declare no conflicts of interest.

## Data Availability

The data that support the findings of this study are available on request from the corresponding author. The data are not publicly available due to privacy or ethical restrictions.

## References

[1] K. S. Muttanahally and A. Tadinada , “A Curious Case Report: Should a Clinician Be Worried About Bone Resorption Under a Chin Augmentation Site?,” Cureus 15, no. 4 (2023): e37041, 10.7759/cureus.37041. 37143644 PMC10154005

[2] P. Van der Geld , P. Oosterveld , G. Van Heck , and A. M. Kuijpers‐Jagtman , “Smile Attractiveness: Self‐Perception and Influence on Personality,” Angle Orthodontist 77, no. 5 (2007): 759–765, 10.2319/082606-349. 17685777

[3] A. Gupta , J. Duhan , and J. Wadhwa , “Prevalence of Three Rooted Permanent Mandibular First Molars in Haryana (North Indian) Population,” Contemporary Clinical Dentistry 8, no. 1 (2017): 38–41, 10.4103/ccd.ccd_699_16. 28566849 PMC5426164

[4] C. P. Klingenberg , M. Barluenga , and A. Meyer , “Shape Analysis of Symmetric Structures: Quantifying Variation Among Individuals and Asymmetry,” Evolution 56, no. 10 (2002): 1909–1920, 10.1111/j.0014-3820.2002.tb00117.x. 12449478

[5] A. Chiu , V. Bertucci , D. D. Coimbra , and D. Li , “Assessment and Treatment Strategies for the Aesthetic Improvement of the Lower Face and Neck,” Clinical, Cosmetic and Investigational Dermatology 16 (2023): 1521–1532, 10.2147/CCID.S405639. 37337568 PMC10276991

[6] B. R. Minehart and J. E. Barrera , “Nuances in Genioplasty,” Facial Plastic Surgery 41, no. 5 (2025): 621–627, 10.1055/a-2626-8646. 40555381

[7] M. Bashour , “An Objective System for Measuring Facial Attractiveness,” Plastic and Reconstructive Surgery 118, no. 3 (2006): 757–776, 10.1097/01.prs.0000207382.60636.1c. 16932187

[8] A. Baus , K. Rem , M. Revol , and S. Cristofari , “Génioplasties d'augmentation prothétiques et osseuses, à visée esthétique: revue de littérature et actualisation des connaissances [Prosthetic Genioplasty versus Osseous Genioplasty in Aesthetic Chin Augmentation: Literature Review And Knowledge Update],” Annales de Chirurgie Plastique et Esthétique 63, no. 3 (2018): 255–261, 10.1016/j.anplas.2017.11.004. 29217082

[9] G. Zammarchi and C. Conversano , “Application of Eye Tracking Technology in Medicine: A Bibliometric Analysis,” Vision (Basel) 5, no. 4 (2021): 56, 10.3390/vision5040056. 34842855 PMC8628933

[10] Z. Cheng , Z. Zhu , W. Yan , C. Zhang , and J. Shi , “A Study of Differences in Eye Tracking and Gaze Patterns of Three Different Surgical Incision Scars for Nipple‐Sparing Mastectomy on Immediate Postoperative Aesthetics of Breast Reconstruction,” Journal of Plastic, Reconstructive & Aesthetic Surgery 104 (2025): 124–131, 10.1016/j.bjps.2025.02.032. 40120220

[11] K. Frank , R. Zeng , S. Sedlbauer , et al., “The Influence of Scar Patterns After Reduction Mammoplasty on Eye Movement and Gaze Pattern: An Eye‐Tracking Investigation,” Aesthetic Plastic Surgery 48, no. 3 (2024): 250–258, 10.1007/s00266-023-03689-1. 37853080 PMC10917861

[12] J. Duan , M. Zhang , M. Song , X. Xu , and H. Lu , “Eye Tracking‐Enhanced Deep Learning for Medical Image Analysis: A Systematic Review on Data Efficiency, Interpretability, and Multimodal Integration,” Bioengineering (Basel) 12, no. 9 (2025): 954, 10.3390/bioengineering12090954. 41007199 PMC12467291

[13] Ž. Pátková , V. Třebický , M. Kocourek , et al., “Visual Attention to Faces During Attractiveness and Dominance Judgements,” Evolutionary Human Sciences 7 (2025): e15, 10.1017/ehs.2025.2. 40337162 PMC12056419

[14] S. von Buelow and N. Pallua , “Efficacy and Safety of Polyacrylamide Hydrogel for Facial Soft‐Tissue Augmentation in a 2‐Year Follow‐Up: A Prospective Multicenter Study for Evaluation of Safety and Aesthetic Results in 101 Patients,” Plastic and Reconstructive Surgery 118, no. 3 Suppl (2006): 85S–91S, 10.1097/01.prs.0000234844.59251.3f. 16936548

[15] J. Z. Lim , J. Mountstephens , and J. Teo , “Emotion Recognition Using Eye‐Tracking: Taxonomy, Review and Current Challenges,” Sensors (Basel) 20, no. 8 (2020): 2384, 10.3390/s20082384. 32331327 PMC7219342

[16] Y. P. Huang and W. R. Li , “Correlation Between Objective and Subjective Evaluation of Profile in Bimaxillary Protrusion Patients After Orthodontic Treatment,” Angle Orthodontist 85, no. 4 (2015): 690–698, 10.2319/070714-476.1. 25347046 PMC8611736

[17] J. M. Sykes , A. Carruthers , B. Hardas , et al., “Development and Validation of a Photonumeric Scale for Assessment of Chin Retrusion,” Dermatologic Surgery 42, no. Suppl 1 (2016): S211–S218, 10.1097/DSS.0000000000000849. 27661743 PMC5671788

[18] J. An , L. Chen , X. Ma , J. Qu , A. Schumacher , and D. Li , “Validation of a Chin Retrusion Scale for Chinese Subjects,” Journal of Craniofacial Surgery 33, no. 1 (2022): 48–51, 10.1097/SCS.0000000000007849. 34260446 PMC8694261

[19] M. S. Lee , D. H. Chung , J. W. Lee , and K. S. Cha , “Assessing Soft‐Tissue Characteristics of Facial Asymmetry With Photographs,” American Journal of Orthodontics and Dentofacial Orthopedics 138, no. 1 (2010): 23–31, 10.1016/j.ajodo.2008.08.029. 20620830

[20] G. Gabriele , N. Pini , S. Cicorella , F. Cascino , V. Ramieri , and P. Gennaro , “The Attractiveness of Facial Profile: A Random Population Survey on the Relationship of Jaws,” Journal of Maxillofacial and Oral Surgery 25 (2024): 421–429, 10.1007/s12663-024-02225-1. 41971483 PMC13065968

[21] D. H. Ajmera , C. Zhang , J. H. H. Ng , et al., “Three‐Dimensional Assessment of Facial Asymmetry in Class III Subjects, Part 2: Evaluating Asymmetry Index and Asymmetry Scores,” Clinical Oral Investigations 27, no. 10 (2023): 5813–5826, 10.1007/s00784-023-05193-x. 37615775 PMC10560190

[22] K. Frank , D. Ehrl , F. Bernardini , et al., “How We Look at Mature Faces: An Eye‐Tracking Investigation Into the Perception of Age,” Aesthetic Surgery Journal 43, no. 2 (2023): 115–122, 10.1093/asj/sjac251. 36099471

[23] J. Kempa , A. Kasielska‐Trojan , B. Antoszewski , et al., “Measuring the Effects of Facial Regional Changes Following Excessive Aesthetic Treatments: A Survey and Eye‐Tracking‐Based Investigation,” Journal of Plastic, Reconstructive & Aesthetic Surgery 105 (2025): 270–280, 10.1016/j.bjps.2025.04.027. 40318363

[24] T. Mashiko , H. Mori , H. Kato , et al., “Semipermanent Volumization by an Absorbable Filler: Onlay Injection Technique to the Bone,” Plastic and Reconstructive Surgery. Global Open 1, no. 1 (2013): e14, 10.1097/GOX.0b013e31828c66b0. PMC4174170 25289198

